# Structural links from trait emotional intelligence to life satisfaction and depressive symptoms in women with breast cancer: post-traumatic responses as mediators

**DOI:** 10.1007/s00737-024-01438-0

**Published:** 2024-01-31

**Authors:** Marco Cannavò, Maria-Jose Sanchez-Ruiz, Nadia Barberis

**Affiliations:** 1 Dipartimento di Scienze della salute, Università degli Studi Magna Graecia di Catanzaro, Viale Europa, 88100 Catanzaro, Italy; 2 Faculty of Education, Universidad de Alcalá de Henares, Pza. San Diego, s/n, 28801 Alcalá de Henares, Madrid Spain

**Keywords:** Breast cancer, Trait emotional intelligence, Post-traumatic stress, Depression, Post-traumatic growth

## Abstract

**Purpose:**

The diagnosis of a disease such as breast cancer (BC) can be experienced as a sudden, unexpected, and life-threatening event accompanied by considerable uncertainty. This experience can precipitate the development of post-traumatic symptoms and depression. Conversely, certain individuals exhibit the capacity to reframe this traumatic event and transform it into an opportunity for personal growth. Existing research shows that individuals with high trait emotional intelligence (trait EI) tend to experience fewer post-traumatic stress symptoms (PTS), and greater post-traumatic growth (PTG). The aim of this study was to investigate the interrelationship among these variables and specifically examine whether PTS and PTG play a mediating role between trait EI, depression, and life satisfaction.

**Methods:**

Questionnaires were administered to 338 women with BC to assess trait EI, PTS, PTG, depression, and life satisfaction.

**Results:**

Results highlighted that trait EI was negatively related to PTS and depression and positively related to PTG and life satisfaction. In addition, both PTS and PTG showed a mediating role in the relationship between trait EI, depression, and life satisfaction. This study highlights the close link between depressive symptoms and post-traumatic cognitions in women with BC.

**Conclusion:**

Current findings highlight links between trait EI, PTS, PTG, depressive symptoms, and life satisfaction. Clinicians could use these findings when developing interventions aimed at alleviating PTS, such as low mood and worry, and facilitating PTG. This study demonstrated that trait EI can reduce PTS and increase PTG, therefore it is important to include programs aimed at fostering trait EI.

Breast cancer (BC) is one of the most frequently diagnosed oncological diseases in women, with an estimated annual 2.3 million new cases (Sung et al. 2021). While BC has recently represented one of the primary causes of oncological mortality in women (Sung et al. 2021), advances in both screening and treatment modalities over the last few decades have considerably improved its prognosis (DeSantis et al. 2019).

Individuals diagnosed with BC often grapple with physical changes, functional limitations, unpleasant side effects, impaired quality of life, and strained interpersonal relationships (Caruso et al. 2017; Hodgkinson et al. 2006). Thus, it is not surprising that they may concurrently experience persistent feelings of sadness and hopelessness, aligning with depressive symptoms. In this regard, a recent meta-analysis by Pilevarzadeh et al. (Pilevarzadeh et al. 2019), which included 72 different studies, highlighted the prevalence of depressive symptoms within this clinical population, revealing a global prevalence of 32.2%.

One important aspect of depressive symptomatology involves forming a pessimistic self-assessment, as well as a negative outlook toward life circumstances and surroundings (Beck 1976; Scher et al. 2004). Similarly, *life satisfaction* may be conceptualized as an overall evaluation of one's life based on self‐selected standards (Diener 1984; Pavot and Diener 2008). In the context of oncological diseases, Guliyeva et al. (2022) demonstrated a connection between lower life satisfaction and a heightened sense of hopelessness. On the other hand, Mostarac and Brajković (2021) underscored that greater life satisfaction can enhance one's perception of life as purposeful and fulfilling, even in the presence of this condition. Well-established literature has confirmed that effectively managing emotions can assist individuals in adapting to challenging conditions such as cancer (Baziliansky and Cohen 2021; Brandão et al. 2016; Carreno et al. 2023). Among emotion-related constructs, the concept of *trait emotional intelligence* (trait EI) has proven to be useful in offering a comprehensive framework for elucidating how individuals generally navigate the complexities of emotional information and respond when confronted with emotionally charged situations (Sarrionandia and Mikolajczak 2019). Specifically, trait EI has been conceptualized as a constellation of emotion-related dispositions positioned at lower levels of personality hierarchies measured via self-report instruments (Petrides et al. 2007). Trait EI encompasses 15 different facets (Table 1) tapping into how individuals perceive themselves in terms of processing emotions both in themselves and others (Petrides et al. 2018).

**Table 1:** Trait emotional intelligence sampling domain

Facet	High scorers view themselves as…
Adaptability	…flexible and willing to adapt to new conditions
Assertiveness	…forthright, frank, and willing to stand up for their rights
Emotion expression	…capable of communicating their feelings to others
Emotion management	(others) …capable of influencing other people’s feelings
Emotion perception	(self and others) …clear about their own and other people’s feelings
Emotion regulation	…capable of controlling their emotions
Impulse control	…reflective and less likely to give in to their urges
Relationships	…capable of maintaining fulfilling personal relationships
Self-esteem	…successful and self-confident
Self-motivation	…driven and unlikely to give up in the face of adversity
Social awareness	…accomplished networkers with superior social skills
Stress management	…capable of withstanding pressure and regulating stress
Trait empathy	…capable of taking someone else’s perspective
Trait happiness	…cheerful and satisfied with their lives
Trait optimism	…confident and likely to “look on the bright side” of life

Accumulating evidence from the clinical field suggests that the concept of trait EI has implications in a wide range of conditions. For instance, the meta-analysis performed by Sarrionandia and Mikolajczak (2019), through the examination of 106 distinct cross-sectional studies, indicated that trait EI plays a pivotal role in predicting both personal perceptions and objective markers of health, underscoring its significance in relation to an individual's overall well-being. In oncological settings, Smith et al. (2012) emphasized that low trait EI predicts increased worry during the early stages of the cancer diagnostic process. Similarly, Chen et al. (2021) demonstrated that, among women with BC, low trait EI was associated with reduced quality of life and increased fear of cancer recurrence or progression.

Not surprisingly, individuals confronted with such a diagnosis may be susceptible to re-experiencing thoughts, nightmares, or other psychological phenomena closely related to their diagnosis. They might also try to avoid stimuli that can trigger memories of their cancer-related experiences and display symptoms such as irritability (Wachen et al. 2014). These manifestations may align with the diagnostic criteria for post-traumatic stress disorder (PTSD; American Psychiatric Association 2013). A recent meta-analysis of 38 studies (Swartzman et al. 2016) examining breast cancer diagnosis and treatment found that the incidence of PTSD symptomatology after this kind of diagnosis is approximately 10%.

One of the pivotal aspects of PTSD symptomatology involves alterations in thoughts and behaviors occurring after exposure to an adverse event (Shalev et al. 2017). Conversely, *post-traumatic growth* (PTG) may be conceptualized as a series of positive changes and beneficial transformations that occur in the aftermath of a traumatic event. PTG serves as a catalyst for individuals to reassess and reconsider their perspective on the world (Tedeschi and Calhoun 2004).

A burgeoning line of empirical studies has shown that one's ability to find new meaning amidst hardship is associated with adaptive coping strategies (Eisenbeck et al. 2021; Sanchez-Ruiz et al. 2021). In the context of BC, Zhai et al. (2019) provided a synthesis of qualitative research on the experience of PTG among women with BC, and four main manifestations and determinants were extracted: a new perception of self, relatedness to others, a new life philosophy, and spiritual and religious growth. Moreover, factors influencing PTG were identified, such as personal characteristics, illness factors, cognitive processing, coping strategies, social support, religion and spirituality, the body’s role, and physical activities.

There is evidence that higher levels of depression occur when *post-traumatic symptomatology* (PTS) is more pronounced (Flory and Yehuda 2022). Since PTS symptomatology implies a sense of hopelessness (Aguglia et al. 2021; Scher et al. 2021), it is reasonable to infer that a higher likelihood of experiencing depressive symptoms follows. Additionally, previous meta-analytic findings (Siddaway et al. 2015) suggest that core beliefs associated with PTS, such as the fear of defeat and entrapment, are also assumed to foster depressive symptoms. On the other hand, considering that satisfaction with life is closely connected to life events (Dore and Bolger 2018; Lazić et al. 2019), it is reasonable to infer that traumatic experiences may reduce one's satisfaction with life. Both PTS and PTG have been linked to individuals' emotional clarity. In this sense, a meta-analysis conducted by Seligowski et al. (2015) summarized the evidence regarding the interplay between PTSD and a wide array of aspects related to emotion regulation. Findings revealed that difficulties in emotion regulation might contribute to post-traumatic symptomatology. In the context of BC, Durosini et al. (2022) conducted a systematic review of 33 studies and emphasized that adaptive emotion management plays a pivotal role in the well-being and health management of BC survivors. Previous research has consistently demonstrated that trait EI enables individuals to perceive potentially overwhelming experiences as challenges rather than threats (Kaliska and Akbey 2019; Mikolajczak and Luminet 2008).

Drawing upon previous research, we expected that individuals with higher trait EI will possess a greater capacity to manage emotionally charged situations, thereby reducing feelings of isolation and inadequacy (Petrides et al. 2018).

In addition, trait EI encompasses emotion-related dispositions that facilitate the management of everyday challenges and the establishment of more profound interpersonal connections. Consequently, it was posited that individuals with high trait EI may experience an enhanced sense of inner resilience and realignment of personal priorities. Conversely, lower levels of trait EI were predicted to lead to heightened PTS and hindered PTG, potentially exacerbating feelings of hopelessness and an inability to effectively cope with feelings of defeat and entrapment. These adverse emotional outcomes, in turn, may contribute to the emergence of depression and increase dissatisfaction with life.

Considering these theoretical and empirical premises, the current study aimed to test the hypothesis that the associations between trait EI, life satisfaction, and depressive symptoms would be mediated by PTS and PTG. Specifically, the goal of this research was to test a model in which greater trait EI predicts PTG that leads in turn to lower depressive symptoms and greater life satisfaction. Conversely, lower trait EI was expected to predict higher PTS symptoms which, in turn, were predicted to promote higher depressive symptoms and lower life satisfaction. It was also expected that higher trait EI would be linked to lower depression and lower life satisfaction.

## Method

### Participants

Questionnaires were administered to 338 women with BC, aged between 25 and 83 years (*M* = 53.36, *SD* = 9.31). The inclusion criteria were a breast cancer diagnosis of at least 1 year, no history of recurrence, and no previous instances of any other type of cancer. Demographics and sample characteristics can be found in Table 2.

**Table 2:** Demographics and sample characteristics

Variables	Percentage (N subjects)
*Educational Qualification*	
University Degree	53% (179)
Post-Graduate Degree	36% (122)
High School Diploma	11% (37)
*Occupational Status*	
Employed	52% (176)
Retired	17% (57)
Housewife	15% (51)
Self-Employed	11% (37)
Unemployed	5% (17)
Student	1% (3)
*Marital Status*	
Married	61% (206)
Divorced	12% (41)
Cohabitating	9% (30)
Single	8% (27)
Engaged	5% (17)
Widowed	4% (14)
*Treatment for breast cancer*	
Tumor removal surgery	95% (321)
Hormone therapy	94% (319)
Chemotherapy	68% (229)
Radiotherapy	64% (216)
More than one treatment	93% (316)

## Measures

### Trait emotional intelligence

The Trait Emotional Intelligence Questionnaire- SF (TEIQue-SF***;*** Petrides 2009) in its Italian validation was used (Di Fabio and Palazzeschi 2011a; 2011b). Detailed instrument description can be found in Table 3.

**Table 3:** Questionnaire description

Variables	Measurement	Definition	Response system	Number of items
1.Trait emotional intelligence	Trait Emotional Intelligence Questionnaire – Short Form (TEIQue-SF)	Self-report questionnaire designed to assess personal levels of trait emotional intelligence (e.g.: "Expressing my emotions with words is not a problem for me"). The questionnaire consists of 4 subscales: Well-Being, Self-Control, Emotionality, and Sociability,	7-point Likert scale from 1 (completely disagree) to 7 (completely agree)	30
2. Post-traumatic growth	Post-traumatic Growth Inventory (PTGI)	Self-report questionnaire designed to assess personal levels of post-traumatic growth (e.g.: “I have a greater appreciation for the value of my own life”). The questionnaire consists of 5 subscales: Personal Strength, New Possibilities, Improved Relationships, Spiritual Growth, and Appreciation for Life	6-point Likert scale from 0 (I did not experience this as a result of my crisis) to 5 (I experienced this change to a very great degree as a result of my crisis)	21
3. Post-traumatic stress disorder	Post-traumatic Stress Disorder Checklist for DSM-5 (PCL-5)	Self-report questionnaire designed to assess personal levels of post-traumatic stress symptoms (e.g.: “In the past month, how much were you bothered by repeated, disturbing, and unwanted memories of the stressful experience?”)	5-point Likert scale from 0 (not at all) to 4 (extremely)	20
4. Depression	Depression scale from Depression Anxiety Stress Scales-21 (DASS-21)	Self-report questionnaire designed to assess symptoms of depression (e.g.:” I couldn't seem to experience any positive feeling at all”)	4-point Likert scale, from 0 (Did not apply to me at all) to 3 (Applied to me very much or most of the time)	7
5. Life satisfaction	Satisfaction with Life Scale (SWLS)	Self-report questionnaire designed to assess levels of satisfaction with life (e.g.: “In most ways my life is close to my ideal”)	7-point Likert scale, from 1 (“Strongly disagree”) to 7 (= “Strongly agree”)	5

### Post-traumatic growth

The Post-traumatic Growth Inventory (PTGI; Tedeschi and Calhoun 1996) in its Italian validation was used (Prati and Pietrantoni 2014). Detailed instrument description can be found in Table 3.

### Post-traumatic stress disorder

The Post-traumatic Stress Disorder Checklist for DSM-5 (PCL-5; Blevins et al. 2015) in its Italian validation was used (Di Tella et al. 2022). Detailed instrument description can be found in Table 3.

### Depression

The Depression Anxiety Stress Scales-21 *(DASS-21;* Lovibond and Lovibond 1995) in its Italian validation was used (Bottesi et al. 2015). Detailed instrument description can be found in Table 3.

### Life satisfaction

The Satisfaction with Life Scale (SWLS; Diener et al. 1985) in its Italian validation were used (Di Fabio and Palazzeschi 2012). Detailed instrument description can be found in Table 3.

### Procedures

For this study, women affected by BC were recruited via social network posts. Participants completed a 35 to 45-min online survey, giving implied consent upon submission. All affirmed their voluntary participation, and anonymity was ensured. To broaden the sample, oncologists collaborated by distributing the survey link to their patients. No monetary compensation was provided to participants or collaborating doctors. Data were collected from 20-Oct-2022 to 20-March-2023.

### Statistical analyses

Correlations and descriptive analyses were conducted for all the observed variables. Collected data were analyzed using IBM SPSS 22 and R. A structural equation modelling with latent variables was used to test a model with trait EI as the predictor variable, PTS and PTG as the mediators, depression and life satisfaction as outcomes.

For the PTG latent construct, the 5 scales that rate the construct were used, while for all the other latent constructs, a parceling approach has been used that consists of the aggregation of items (randomly selected) from the scales in three indicators of each latent variable (Little et al. 2002). Parcels are more likely to meet the assumptions of normality and less likely to be influenced by method effects (Little et al. 2002). The *Lavaan* Package for R with the integration of RStudio was used to perform the analysis of the covariance matrices, and solutions were generated based on maximum-likelihood estimation. The significance of the indirect effects that emerged (i.e., the drop from the total to direct effect) was explored using the bootstrap-generated bias-corrected confidence interval approach (Preacher and Hayes 2004; Shrout and Bolger 2002).

## Results

### Descriptive results and correlations

Means, Standard Deviations, Skewness, and Kurtosis of scores of each variable are shown in Table 4. Furthermore, Table 4 illustrates the correlations among the variables observed and the internal consistency values of each questionnaire.

**Table 4:** Descriptive and correlation analyses

	α	M	SD	Skew	Kurt	1	2	3	4	5	6	7	8	9
1. Trait emotional intelligence	.88	5.01	.78	-.35	-.34									
2. Post-traumatic symptoms	.92	2.42	.85	.36	-.62	-.42**								
3. Personal strength	.82	3.94	.93	-1.27	2.09	.44**	-.17**							
4. New possibilities	.85	3.62	1.05	-.88	.60	.41**	-.12*	.74**						
5. Improved relationships	.87	3.40	1.06	-.60	-.09	.38**	-.16**	.59*	.65**					
6. Spiritual growth	.78	2.81	1.59	-.30	-1.00	.19**	.00	.38**	.45**	.50**				
7. Appreciation for life	.80	4.21	.89	-1.37	2.25	.30**	.01	.64**	.67**	.53**	.38**			
8. Post-traumatic growth	.93	3.62	.87	-.72	.49	.44**	-.13*	.82**	.88**	.88**	.64**	.75**		
9. Life satisfaction	.92	4.33	1.48	-.34	-60	.53**	-.40**	.35**	.41**	.46**	.28**	.29**	.47**	
10. Depression	.90	.94	.78	.80	-.21	-.60**	.71**	-.35**	-.30**	-.28**	-.13*	-.22**	-.33**	-.49**

Note: * p < .05, ** p < .01

### Mediation model

**Fig. 1:**
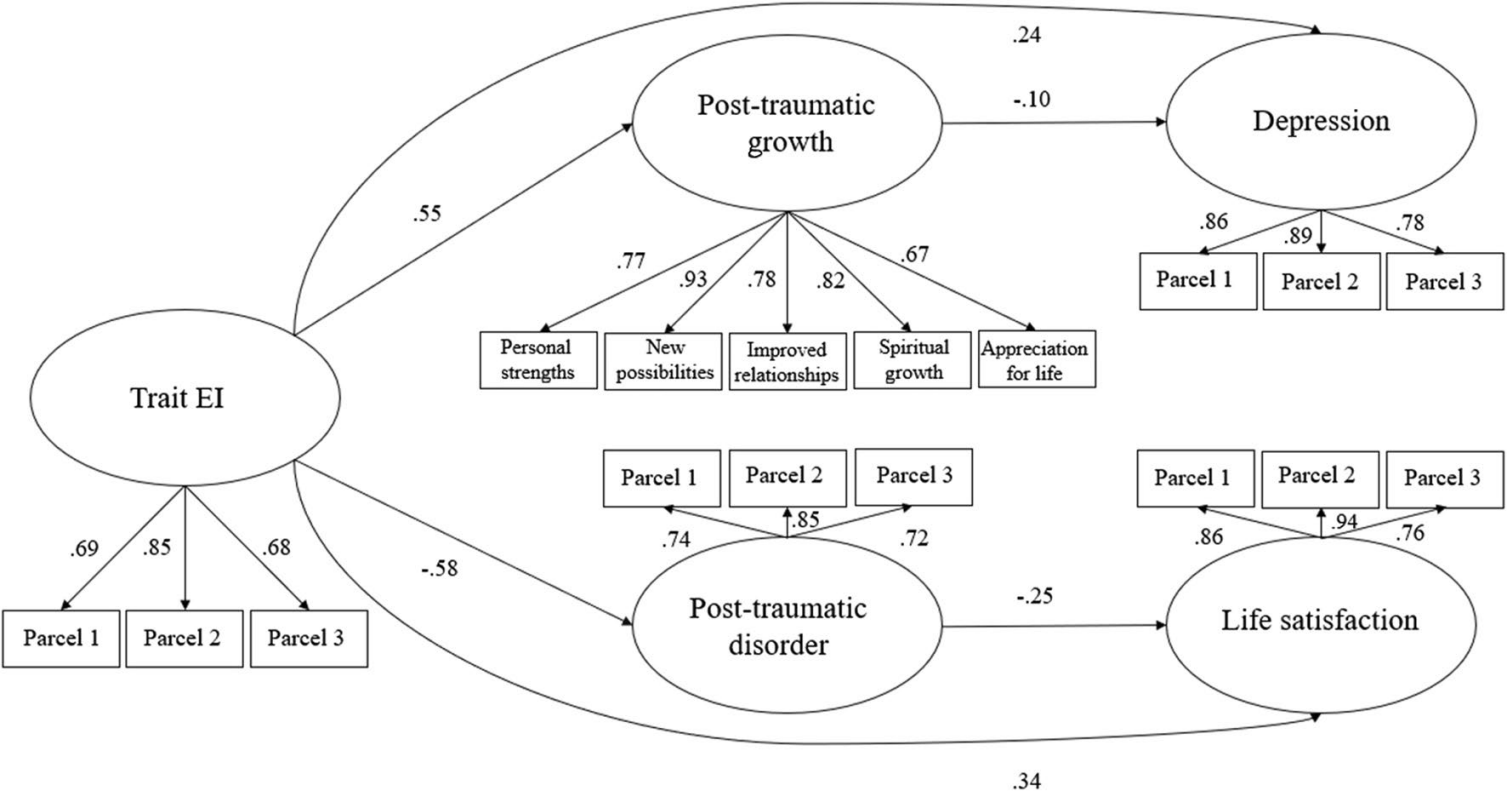
Mediation model between trait EI, PTS, PTG, depressive symptoms, and life satisfaction. Note: Circles represent the latent variables, and boxes represent the observed variables. Coefficients shown are standardized path coefficients

The model showed acceptable fit indices: *χ*2(110) = 243.80; *p* < 0.001, CFI = 0.97, RMSEA = 0.06 (90% CI = 0.05 – 0.07), SRMR = 0.06 (Fig. 1). A detailed description of path estimates can be found in Table 3. Significant paths were found from trait EI to PTS (*β* = -0.54), PTG (β = 0.50), depression (β = -0.24), and life satisfaction (β = 0.34). Moreover, significant paths were found from PTS to depression (β = 0.68), and life satisfaction (β = -0.25). Furthermore, significant paths were found from PTG to depression (β = -0.11), and life satisfaction (β = 0.26). A statistically significant indirect association was found from trait EI to depression (β = -0.37), and life satisfaction (β = 0.14) by PTS. Moreover, indirect associations were found from trait EI to depression (β = -0.06), and life satisfaction (β = 0.13) by PTG (Table 5).

**Table 5:** Path estimates, SEs, and 95% CIs for models with direct effect and indirect effect

	b	Lower bound (BC)	Upper bound (BC)	β
		95% CI	95% CI	
*Direct Effects*				
Trait EI → Post traumatic growth	.55	.42	.69	.50
Trait EI → Post traumatic symptoms	-.58	-.72	-.45	-.54
Trait EI → Life satisfaction	.65	.40	.90	.34
Trait EI → Depression	-.25	-.36	-.14	-.24
Post traumatic growth → Life satisfaction	.44	.25	.64	.26
Post traumatic growth → Depression	-.10	-.18	-.02	-.11
Post traumatic symptoms → Life satisfaction	-.44	-.65	-.23	-.25
Post traumatic symptoms → Depression	.66	.53	.77	.68
*Indirect Effects via Post-traumatic symptoms*				
Trait EI → Life satisfaction	.26	.13	.39	.31
Trait EI → Depression	-.38	-.48	-.29	-.37
*Indirect Effects via Post-traumatic growth*				
Trait EI → Life satisfaction	.25	.13	.36	-.06
Trait EI → Depression	-.06	-.10	-.01	-.06

Note: SE = standards errors; BC 95% CI = Bias Corrected-Confidence Interval

## Discussion

The present study sought to observe whether PTG and PTS would mediate the associations between trait EI, life satisfaction, and depressive symptoms. As expected, results clearly showed that low trait EI was linked to depressive symptomatology by a direct and indirect association (by PTS). These results endorse the idea that enhanced emotional processing and regulation are associated with increased psychological well-being (Martins et al. 2010). In fact, trait EI may assist individuals in better managing their feelings of loneliness and hopelessness; consequently, they can better address any concerns related to their own self-worth, resulting in fewer negative self-evaluations.

When high trait EI scorers are faced with stressful events, they might be more likely to adaptively evaluate their emotional and physiological responses, which helps in maintaining joy and reducing feelings of sadness, shame, and anger (Mikolajczak and Luminet 2008). Such an adaptive evaluation process may, in turn, positively influence the encoding of emotionally charged memories and the processing of cancer-related beliefs. These findings align with those of Kaliska and Akbey (2019), who showed that trait EI can mitigate the adverse effects of exposure to traumatic events and also accord well with research conducted by Chen et al. (2021), who reported that trait EI might help counteract maladaptive thought patterns, such as the fear of cancer recurrence or progression, in women with BC.

In addition, individuals with high trait EI often utilize effective emotional regulation strategies, enabling them to flexibly manage their emotions as needed (Peña-Sarrionandia et al. 2015). This flexibility may enhance their capacity to cope with the unpleasant emotional states that arise from very challenging events, such as a BC diagnosis. Consequently, this adaptive response may reduce their perception of lacking control or influence over their circumstances, ultimately leading to more positive self-evaluations and inner strength. Overall, these insights resonate with those provided by Durosini et al. (2022), whose systematic review found that the adaptive handling of emotions improves the overall quality of life in women with BC. Likewise, our findings point out the link between trait EI and life satisfaction through both a direct and an indirect pathway (mediated by PTS symptoms) in patients with the same condition.

As emphasized by Petrides et al. (2016), those with high trait EI may employ face-to face interactions to become fully adapted to the context (Baudry et al. 2018; Cannavò et al. 2023), culminating in a positive life evaluation. The findings also align with accumulating evidence suggesting that trait EI may be a key dimension influencing varying levels of psychophysical well-being in the context of organic diseases (Barberis et al. 2016, 2022, 2023a, b, c; Sarrionandia and Mikolajczak 2019).

Consistent with the study hypothesis, the current study findings highlight that when women with BC have higher trait EI, they seem to experience a high level of PTG. Individuals with high trait EI scores are more likely to perceive problematic situations as challenges rather than threats (Schneider et al. 2013). This tendency may help them in adopting adaptive strategies during their recovery, such as seeking help from significant others, practicing self-care, and adhering optimally to medical treatments. In turn, this can lead to a sense of personal growth following a traumatic event (Tuck and Patlamazoglou 2019). Furthermore, it was also found that greater trait EI is associated (indirectly, through increased PTG) with lower depressive symptoms and higher life satisfaction. This connection can be explained by the idea that individuals with higher trait EI possess the ability to effectively manage emotionally charged information in both intrapersonal and social contexts, and they effectively utilize face-to-face interactions when dealing with difficult situations (Petrides et al. 2016, 2018). Consequently, they may be more inclined to share their feelings and experiences with others, which can facilitate the process of finding new meaning in a difficult experience. This, in turn, may foster adaptive thinking patterns concerning oneself, the world, and the future, ultimately resulting in higher life satisfaction and lower degrees of depression. This is consistent with the review carried out by Zhai et al. (2019) that suggested that relatedness and connection with others may constitute a determinant of PTG in women with BC. One noteworthy finding of this study is the negative association between components of PTG and PTS. When Post-Traumatic Stress (PTS) becomes invalidating, it may hinder cognitive processing needed for growth (Zebrack et al. 2014), suggesting that increased PTS makes growth less likely. One of the limitations of the present study is the cross-sectional nature of the data, which does not allow for causal inferences concerning the hypothesized relations. Moreover, the exclusive use of self-report measures to assess psychopathological dimensions can lead to measurement bias. Future researchers may want to consider both longitudinal designs and a multi-method approach to data collection, as well as sources such as clinical interviews. It is worth noting that most participants in the study were highly educated, employed, and engaged in various forms of relationships. Abundant research indicated that a higher socioeconomic status may serve as a protective factor against depressive symptoms (Lasserre et al. 2022), while others have emphasized the crucial role of social support in fostering higher PTG (Henson et al. 2021). Future studies should replicate the current study design with more diverse samples, including individuals with lower socioeconomic status, to validate the present findings. Despite these limitations, findings from this study provide several practical insights that clinicians may find useful. More precisely, this study highlights the close link between post-traumatic cognitions and depressive symptoms in women with BC. Consequently, it would be beneficial to consistently screen this clinical population to prevent potential complications. Furthermore, clinicians may consider both addressing PTS in their patients as part of their interventions and recommending PTG aspects such as promoting reframing of one’s experience and fostering personal resources like self-care practices and resilience to reduce low mood and worry. Moreover, the present study highlights how low trait EI in women with BC is closely linked to psychological maladjustment. Given that a wide body of studies highlighted that trait EI can improve via targeted interventions (Hodzic et al. 2018), practitioners could facilitate programs aimed at fostering this set of emotional self-perceptions. Previous interventions (Hodzic et al. 2018; Nelis et al. 2009) have demonstrated that focusing on aspects such as labeling and acknowledging emotions, emotional facilitation of thinking, and reflective regulation of emotion was effective in enhancing individuals’ levels of trait EI. Therefore, incorporating modules that target these facets may enhance treatment efficacy.

## Data Availability

The data that support the findings of this study are available from the corresponding author.
